# Determinants and prognostic value of blood pressure trajectories during graded exercise

**DOI:** 10.1007/s00421-026-06258-x

**Published:** 2026-05-08

**Authors:** Nicholas Cauwenberghs, Anna Carlén, Thomas Lindow, Viktor Elmberg, Lars Brudin, Magnus Ekström, Kristofer Hedman

**Affiliations:** 1 Research Unit Hypertension and Cardiovascular Epidemiology, Department of Cardiovascular Sciences, KU Leuven, Leuven, Belgium; 2 Department of Health, Medicine and Caring Sciences, Linköping University, Universitetssjukhuset, 581 85 Linköping, Sweden; 3 Clinical Department of Clinical Physiology, in Linköping, Region Östergötland, Linköping, Sweden; 4 Department of Physical Education and Sports, Sport and Health University Research Institute (iMUDS), University of Granada, Granada, Spain; 5 Department of Medicine, Växjö Central Hospital, Region Kronoberg, and Clinical Sciences, Pulmonary Medicine, Allergology and Palliative Medicine, Lund University, Lund, Sweden; 6 Department of Research and Development, Växjö Central Hospital, Region Kronoberg, and Clinical Sciences, Pulmonary Medicine, Allergology and Palliative Medicine, Lund University, Lund, Sweden; 7 Department of Clinical Sciences Lund, Respiratory Medicine, Allergology and Palliative Medicine, Faculty of Medicine, Lund University, Lund, Sweden; 8 Department of Clinical Physiology, Blekinge Hospital, Karlskrona, Sweden; 9 Department of Clinical Physiology, Kalmar County Hospital, Kalmar, Sweden

**Keywords:** Blood pressure determination, Exercise test, Time factors, Cardiovascular risk

## Abstract

**Purpose:**

The systolic blood pressure (SBP) response to exercise reflects cardiovascular health, with both exaggerated and blunted responses linked to adverse outcomes. Prior studies relied typically on peak SBP or two-point slopes for evaluation. We aimed to identify distinct SBP responses from full times-series recorded during maximal, graded exercise and assess their clinical determinants and prognostic relevance.

**Methods:**

In this cohort study, we analysed SBP recordings from 6107 patients (mean age, 55.4 years; 45% women) who underwent maximal cycle ergometry. Group-based trajectory modelling (GBTM) extracted sex-specific responses from absolute SBP and relative change (∆SBP) traces. Associations with clinical factors and incident major adverse cardiovascular events (MACE) were assessed using ordinal logistic regression and Cox survival analyses, respectively.

**Results:**

Per sex, GBTM identified four SBP and four ∆SBP trajectories, with weak overlap between them. Higher SBP and lower ∆SBP trajectories were associated with adverse clinical profiles, including higher age and lower exercise capacity. MACE incidence was highest in the high SBP and low ∆SBP groups, but these response categories did not independently predict MACE after adjustment for resting SBP. Among patients with normotension at rest, however, a high SBP response (along with a low ∆SBP in men) was independently associated with increased MACE risk. Patients presenting both high SBP and low ∆SBP consistently conferred the highest MACE risk.

**Conclusions:**

Time-series analysis of SBP measurements during graded exercise revealed distinct response patterns with value for MACE prediction, particularly in normotensive individuals. Integrating such analyses into exercise testing may refine cardiovascular risk stratification.

**Graphical abstract:**

Identification and validation of trajectory-based systolic blood pressure (SBP) responses from time-series recorded during maximal graded exercise

**Figure:**
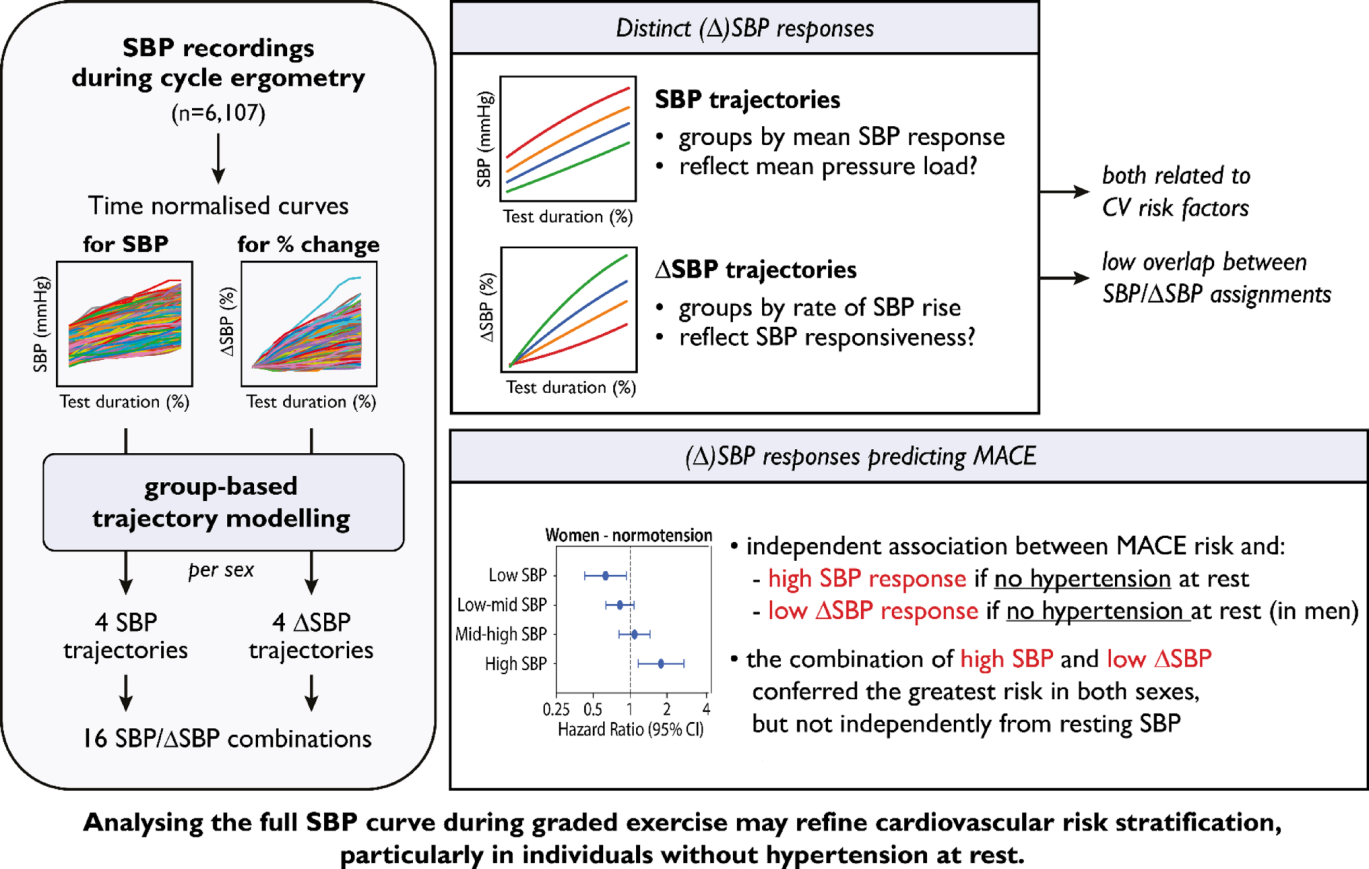

**Supplementary Information:**

The online version contains supplementary material available at 10.1007/s00421-026-06258-x.

## Introduction

Systolic blood pressure (SBP) is a standard measure during clinical exercise testing, providing key information on hemodynamic adaptation to stress and overall cardiovascular health (Pesova et al. 2023). Under normal physiological conditions, SBP rises proportionally with increasing workload, reflecting the rise in cardiac output required to meet higher metabolic demands (Rowell 1993). Both an exaggerated and a blunted SBP response have been linked to adverse outcomes (Barlow et al. 2014; Carlén et al. 2024; Zafrir et al. 2022; Miyai et al. 2000; Schultz et al. 2013). A drop in SBP, typically more than 10 mmHg, is a well-established marker of poor cardiovascular prognosis (Barlow et al. 2014). Likewise, a failure to increase SBP may indicate underlying pathology, such as left ventricular dysfunction or ischemic heart disease (Fletcher et al. 2001). Conversely, an exaggerated SBP response was found to increase the risk of masked and future hypertension and overall cardiovascular risk (Carlén et al. 2024; Zafrir et al. 2022; Miyai et al. 2000; Schultz et al. 2013; Benbassat 1986).

Previous studies have primarily investigated discrete markers from the exercise SBP response, including peak SBP values (Hedman et al. 2022) or linear slopes between two measurement points (Hedman et al. 2019; Carlén et al. 2025). While informative, these simplified metrics capture only a fraction of the SBP response. Extracting information from the entire SBP trajectory, from rest to maximal exertion and anything in between, may bring deeper understanding of blood pressure regulation during exercise. In particular, integrating both absolute SBP values and their temporal progression may provide additional physiological and prognostic insights beyond traditional single-point or two-point measures.

Within this context, trajectory modelling may objectively characterize integrative SBP response patterns during exercise. These algorithmic methods enable unbiased analysis of time-series data (Nguena Nguefack et al. 2020; Magrini 2022), facilitating the identification of distinct groups defined by their absolute or relative SBP dynamics across the entire exercise protocol instead of relying on isolated measurements. By incorporating repeated SBP measurements, trajectory modelling could reduce reliance on single values that may be sensitive to random variability or measurement error and may allow a more granular phenotyping of exercise SBP responses beyond the binary classification obtained by traditional SBP peak or change cutoffs.

For instance, Nene et al. recently identified three SBP trajectories based on absolute SBP values recorded during the initial stages of a Bruce treadmill protocol, which were predictive for future major adverse cardiovascular events (MACE) in unadjusted analyses (Nene et al. 2025). Yet, those trajectories were not sex specific and were derived from a protocol with only three workload stages, limiting analyses to exercise intensities within a (for many patients) submaximal workload range. In contrast, exercise testing with a ramp protocol applies gradual incremental increases in workload, producing smoother and potentially physiologically more informative SBP curves that extend until maximal exertion is reached. This design enables assessment of the SBP response across relative exercise intensities, from rest to peak effort, rather than at a few fixed workload levels. To date, however, the physiological and clinical significance of such integrative SBP responses to maximal graded exercise remains unexplored. Moreover, it is unknown whether combining information from absolute and relative SBP trajectories could enhance cardiovascular risk prediction.

We therefore aimed to algorithmically identify distinct absolute and relative SBP responses from full time-series recorded during maximal graded cycle ergometry and assess their clinical determinants and relevance for MACE prediction.

## Methods

### Study cohort

The cohort study was approved by the Regional Ethical Review Board (2012/379-31 and 2018/141-31). Individual consent was waived given the use of given the use of retrospective and large-scale pseudonymized research data, presented here only at a group level. We used a retrospective clinical database comprising graded exercise tests of 14,401 unique patients (Supplemental Fig. 1) (Hedman et al. 2021; Lindow et al. 2020). We excluded non-bicycle protocols (n = 643), tests performed by minors (n = 622) and submaximal tests, defined either as a test under 6 min (n = 2,036) or as a rate of perceived exertion under 17 or missing (n = 1,234). Additionally, 3,025 tests were removed as the SBP time-series did not meet the strict quality criteria required for reliable assessment of SBP trajectories. Among others, these criteria included fewer than four SBP measurements during exercise or a first SBP value obtained more than 200 s after exercise initiation (i.e. 180 s to initiate SBP measurement + 20 s margin for registration). Furthermore, we removed patients with documented valvular heart disease (n = 143), pacemaker or implantable defibrillator (n = 2) and those at high risk for inaccurate SBP measurements due to class III obesity (body mass index (BMI) ≥ 40 kg/m^2^) (n = 163) or the presence of arrhythmia during the test (n = 412). An additional 14 people were excluded due to insufficient quality of the SBP traces noticed during visual review, yielding a total analysable sample of 6,107 patients (Supplemental Fig. 1).

### Graded exercise testing

All patients underwent a ramp protocol on an electrically braked cycle ergometer (Rodby Inc, Karlskoga, Sweden) (Lindow et al. 2020). Most tests started at 50 Watts in men and 30 Watts in women, followed by workload increments of 15 Watts/min and 10 Watts/min respectively, until maximal volitional exertion. In line with clinical recommendations, the target exercise duration was 8 to 12 min. SBP was measured at rest and subsequently every 2–3 min during exercise. In line with best practice recommendations for exercise BP measurements (Schultz et al. 2022), SBP was monitored through manual auscultation, using a Doppler probe over the radial artery of the right arm. From the achieved peak workload, the percentage of the predicted workload (ppWL) was calculated, considering age, sex and height (Brudin et al. 2014). ppWL ≤ 75% indicated impaired exercise capacity. Subsequently, time-stamped SBP measurements were exported for analysis. An exaggerated SBP response was defined as peak SBP > 210 mmHg in men and > 190 mmHg in women.

### Medical data

Information on inpatient and outpatient diagnoses, dispensed outpatient medication and mortality was retrieved from Swedish national health registries, as described previously (Hedman et al. 2021; Lindow et al. 2020). Data on cause and date of death were obtained from the Swedish Causes of Death Register, which is considered highly complete and reliable (cut-off: 30 April 2019) (Brooke et al. 2017). From the medical data, MACE was defined as cardiovascular death, ischemic heart disease, stroke or heart failure occurring after the exercise test. Only the first event was considered in survival analyses.

### Data management and statistical analysis

**Fig. 1:**
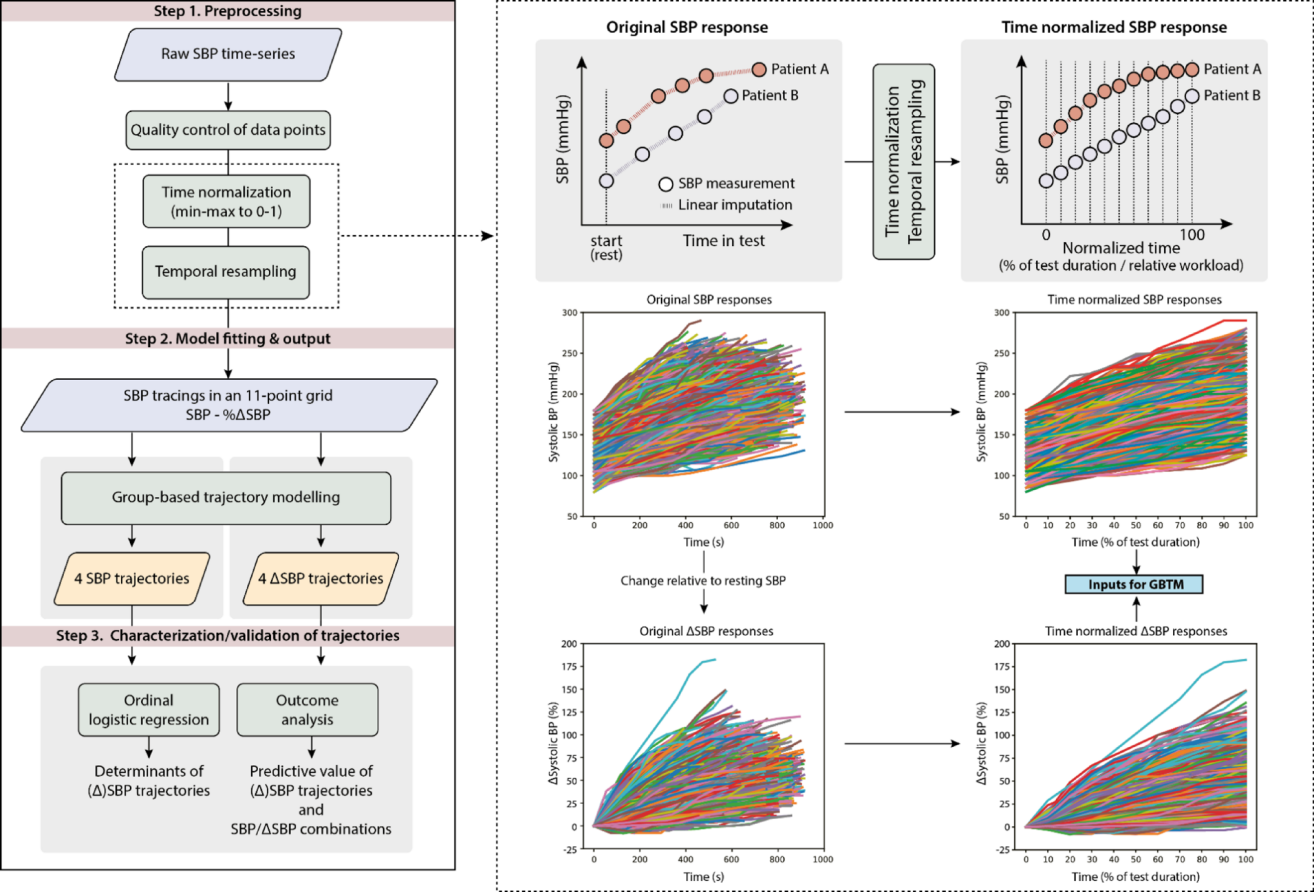
Analytical workflow to derive and validate exercise SBP trajectories. Rhomboids represent input and output data. Rectangles indicate analytical steps. *GBTM* group-based trajectory modelling, *SBP* systolic blood pressure.

Data processing and analyses were performed in Python 3.14 using common data science libraries. Figure 1 presents the three analytical steps to identify and validate exercise SBP trajectories from the series of absolute SBP values or the percentage change between SBP at each time point and resting SBP (i.e. the relative changes in SBP, ∆SBP). For this, we aimed to differentiate between response profiles based on the average SBP response (largely determined by mean exercise SBP) and profiles based on the SBP dynamics (largely reflected by the slope or relative rise in SBP).

*Step 1: Preprocessing of SBP time-series.* As described for this cohort elsewhere (Cauwenberghs et al. 2026), we applied a heuristic, rule-based quality control procedure to identify implausible data points within the SBP time-series. First, SBP recordings were omitted if they did not pass the general technical and physiological checkpoints (Supplemental Fig. 1). Next, abrupt changes between consecutive exercise SBP measurements were benchmarked to physiological expectations and flagged for visual review if they exceeded a > 20 mmHg decrease or > 60 mmHg increase. Of 36 flagged time-series, we omitted 14 time-series due to overall poor quality and removed 10 implausible/disruptive data points that were likely attributable to measurement errors or recording mistakes.

If resting seated SBP was more than 10 mmHg higher than the first exercise SBP (as was the case for 379 patients), the resting SBP was adjusted to equal the first exercise SBP + 10 mmHg. This adjustment minimized the influence of anticipatory responses (e.g. anxiety before the exercise test) that could otherwise cause artefactual declines in SBP at the beginning of the test that would disproportionally affect trajectory modelling. For the 6,107 quality-approved tests, the median number of SBP measurements, including resting SBP, was 6 (10th to 90th percentile: 5 to 8).

For standardization, each patient’s exercise duration was then rescaled to a 0–100% range using min–max normalization (Fig. 1). This normalization enabled comparison of SBP responses across tests of varying duration. Because workload increased gradually and linearly, normalization by test time or workload was effectively equivalent, as reflected by the near-perfect within-patient correlation between these axes (r = 0.99 ± 0.01). Next, (∆)SBP values were linearly interpolated to generate evenly spaced 11-point SBP traces, corresponding to one data point per 10% of test duration (Fig. 1).

*Step 2: Group-based trajectory modelling SBP trajectories.* GBTM is a statistical method used to identify groups within a population that show a distinct pattern of change over time. Instead of relying on a single measurement, GBTM thus groups individuals that present a similar trend in a variable over time (Nguena Nguefack et al. 2020). In this study, GBTM was employed to identify distinct exercise-related (∆)SBP trajectories based on the full SBP time-series, where each trajectory groups patients with a distinct (∆)SBP response. GBTM was conducted separately on either absolute SBP and ∆SBP tracings, using the R package ‘gbmt’ (v0.1.3) (Magrini 2022), with normalized test duration as the time scale. GBTM was conducted for men and women separately to avoid sex bias in the trajectory assignments due to well-characterized sex differences in the SBP response to exercise (Gleim et al. 1991; Li et al. 2025). GBTM models with two to ten quadratic trajectories were evaluated and, in both men and women and for both SBP and ∆SBP, the Bayesian information criterion (BIC) indicated that four-trajectory models provided the best fit overall (Supplemental Fig. 2).

Accordingly, each patient was assigned to one of the four trajectory groups for SBP and for ∆SBP for which they had the greatest posterior predictive probability. Additionally, the four SBP trajectories were qualitatively labelled ‘low SBP’, ‘low-mid SBP’, ‘mid-high SBP’ and ‘high SBP’, matching their visual appearance. Analogously, the four ∆SBP trajectories were qualitatively labelled ‘low SBP rise, ‘low-mid SBP rise’, ‘mid-high SBP rise’ and ‘high SBP rise’. In exploratory analyses, trajectories for absolute and relative ∆SBP were strongly overlapping. We opted for the relative ∆SBP trajectories because of the inherent standardization for resting SBP.

*Step 3: Validation of exercise (∆)SBP trajectories.* To evaluate the clinical relevance of the exercise (∆)SBP groups, we analysed their associations with baseline clinical factors and MACE risk. Of note, the extracted ∆SBP groups should be interpreted as a standardized descriptor of SBP dynamics and do not represent a direct physiological measure of pressure load or a replacement for absolute SBP change.

First, we quantified overlap in peak SBP distributions across trajectory groups using the overlapping coefficient derived from kernel density estimates. Agreement between trajectory-based and peak SBP classifications (non-exaggerated vs. exaggerated SBP response) was further assessed using cross-tabulation. Next, clinical characteristics were compared across groups using pairwise Z-tests. Ordinal logistic regression was applied to estimate the multivariable-adjusted odds of assignment to a higher trajectory category (e.g. moving from ‘low SBP’ to ‘low-mid SBP’), adjusting for age, BMI, hypertension, antihypertensive medication, use of beta blockers, lipid-lowering drugs, anticoagulants, diabetes mellitus, cardiac disease, resting heart rate and ppWL.

For survival analyses, Kaplan–Meier curves were generated for each (∆)SBP trajectory category, with log-rank tests to compare event-free survival between groups. Cox proportional hazards models were fitted to calculate hazard ratios (HR) for MACE relative to the cohort’s average risk. Adjusted HRs accounted for differences in significant predictors of MACE: baseline age, cardiac disease, chronic obstructive pulmonary disease, diabetes and the use of antihypertensive medication (excluding beta blockers), beta blocker use, lipid-lowering drugs, anticoagulants and ppWL (impaired vs normal exercise capacity). Additionally, we tested the impact of accounting for resting SBP. To explore the clinical value of our methodology beyond traditional hypertension assessment, we repeated the Cox modelling by hypertensive status at rest (i.e. those with resting SBP ≥ 140 mmHg, resting DBP ≥ 90 mmHg or prior clinical diagnosis of hypertension versus normotensive counterparts). Prior to Cox modelling, we verified that no problematic multicollinearity was present among the covariates by evaluating their variance inflation factors (≤ 3.12 for all).

Next, we examined whether SBP and ∆SBP trajectories may provide complementary information. For this, we first evaluated concordance between the SBP and ∆SBP trajectory assignments using Cramer’s V, a measure of association between two categorical variables. We then repeated the Cox modelling for the sixteen combinations in SBP/∆SBP trajectories (e.g. low SBP/low SBP risers).

## Results

### Characterization of exercise (∆)SBP groups

**Fig. 2:**
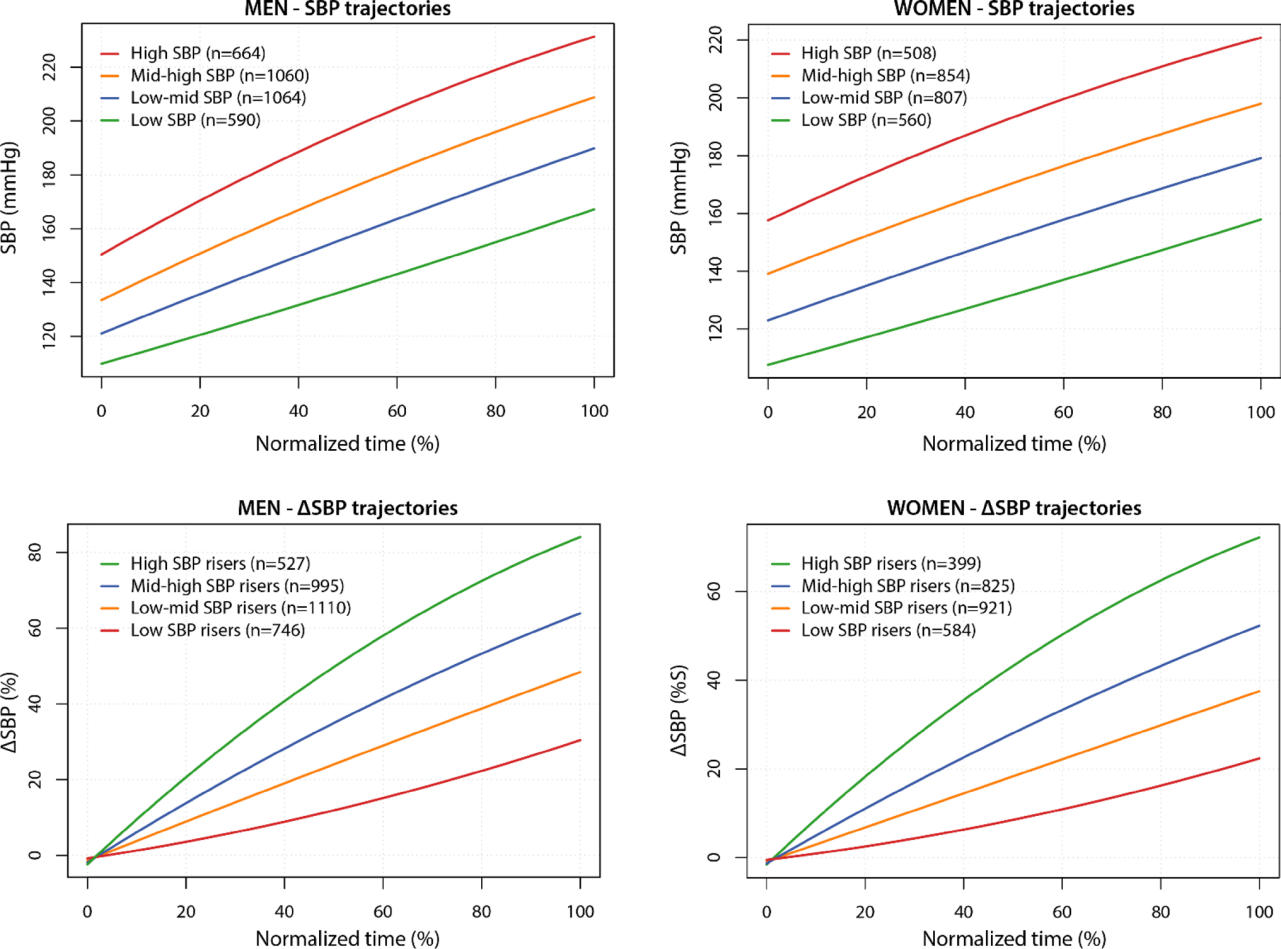
Exercise SBP trajectories derived by group-based trajectory modelling (GBTM). GBTM identified four distinct response profiles from the SBP and ∆SBP time-series in men and women. Each panel shows the regression line for each trajectory. The profiles were labelled to match their physiological appearance.

Supplemental Table 1 characterizes the cohort of 6,107 patients (mean age, 55.4 years, 45% women). In both sexes, GBTM identified four distinct trajectories based on either absolute SBP values or ∆SBP, corresponding to low, low–mid, mid–high, and high responses (Fig. 2). For both absolute SBP and ∆SBP, the low-mid and mid-high groups were most prevalent, whereas the high groups were least common (Fig. 2). Supplemental Figs. 3 and 4 show the individual ∆SBP time-series assigned to each trajectory. Importantly, group assignment was not determined solely by peak (Δ)SBP but rather by the full (Δ)SBP time-series. For instance, individuals with similar peak values could be classified into different trajectories as indicated by a substantial overlap between peak SBP across the SBP groups (Supplemental Fig. 5A) and between peak ΔSBP distributions across the peak ΔSBP response groups (Supplemental Fig. 5B). Supplemental Table 2 shows the agreement between the (Δ)SBP response groups and exaggerated SBP response (peak SBP > 210 mmHg in men, > 190 mmHg in women).

Clinical characteristics by SBP and ∆SBP group are summarized in Supplemental Tables 3 and 4. Overall, age, BMI, prevalence of hypertension and use of antihypertensive and lipid-lowering medication increased significantly from the low to high SBP group and from the high to low ∆SBP group. In addition, both low SBP and low ∆SBP responders exhibited the lowest ppWL.

### Clinical determinants of exercise (∆)SBP groups

Multivariable ordinal logistic regression identified several independent predictors of trajectory assignment (Table 1). Assignment to a higher SBP group was associated with older age, higher BMI, hypertension, higher resting heart rate and the absence of cardiac disease or anticoagulants (*P* ≤ 0.047 for all). In men, the absence of beta blockers additionally predicted higher SBP response. In contrast, assignment to a higher ∆SBP group was independently associated with younger age, lipid-lowering therapy, and the absence of beta blockers (*P* ≤ 0.017 for all). Additional predictors for higher ∆SBP group were resting heart rate in men and normotension and diabetes mellitus in women. Notably, in both sexes, the assignment to a higher SBP and higher ∆SBP group was independently related to a higher ppWL (Table 1).

**Table 1:** Clinical correlates of SBP and ∆SBP trajectories in multivariable ordinal logistic regression by sex

Variable	SBP trajectories (from low to high SBP)				∆SBP trajectories (from low to high risers)			
	Men		Women		Men		Women	
	OR (95% CI)	*P* value	OR (95% CI)	*P* value	OR (95% CI)	*P* value	OR (95% CI)	*P* value
Age, + 1 SD	1.54 (1.43–1.65)	< 0.001	2.30 (2.12–2.50)	< 0.001	0.67 (0.62–0.72)	< 0.001	0.77 (0.71–0.83)	< 0.001
Body mass index, +1 SD	1.27 (1.19–1.35)	< 0.001	1.35 (1.26–1.45)	< 0.001	0.95 (0.89–1.01)	0.084	0.97 (0.90–1.04)	0.37
Hypertension, yes vs no	2.01 (1.70–2.38)	0.035	2.04 (1.71–2.44)	< 0.001	0.93 (0.79–1.09)	0.37	0.64 (0.54–0.77)	< 0.001
Use of beta blockers, yes vs no	0.74 (0.61–0.91)	0.004	0.94 (0.77–1.15)	0.54	0.51 (0.42–0.63)	< 0.001	0.44 (0.36–0.54)	< 0.001
Lipid-lowering drugs, yes vs no	0.99 (0.80–1.23)	0.95	0.94 (0.74–1.20)	0.62	1.32 (1.06–1.64)	0.012	1.35 (1.06–1.72)	0.017
Anticoagulants, yes vs no	0.56 (0.44–0.70)	< 0.001	0.72 (0.57–0.91)	0.006	0.97 (0.77–1.22)	0.81	0.93 (0.73–1.18)	0.55
Diabetes mellitus, yes vs no	1.05 (0.80–1.38)	0.72	1.30 (0.91–1.87)	0.15	0.85 (0.65–1.12)	0.25	2.09 (1.46–2.99)	< 0.001
Cardiac disease, yes vs no	0.79 (0.63–0.98)	0.030	0.79 (0.63–1.00)	0.047	0.89 (0.72–1.11)	0.31	1.00 (0.79–1.26)	0.98
Resting heart rate, + 1SD	1.12 (1.05–1.19)	< 0.001	1.21 (1.13–1.31)	< 0.001	0.92 (0.86–0.99)	0.017	1.04 (0.97–1.12)	0.30
ppWL, + 1SD	1.46 (1.37–1.56)	< 0.001	1.10 (1.02–1.18)	0.009	1.36 (1.27–1.45)	< 0.001	1.35 (1.25–1.45)	< 0.001

Odds ratios indicate the odds to move one ∆SBP trajectory up (e.g. move from low to low-mid SBP) per standard deviation increase in the predictor variable (if continuous) or in the presence of the predictor variable (if categorical). ppWL indicates percentage predicted workload.

### Exercise (∆)SBP groups and MACE risk

During a median follow-up of 7.1 years [5–95th percentile: 1.6 to 12.9 years] in men and 7.6 years [3.3 to 13.0 years] in women, 1,167 patients (19.1%) experienced a MACE. Supplemental Table 5 shows the event rates by SBP and ∆SBP groups.

**Fig. 3:**
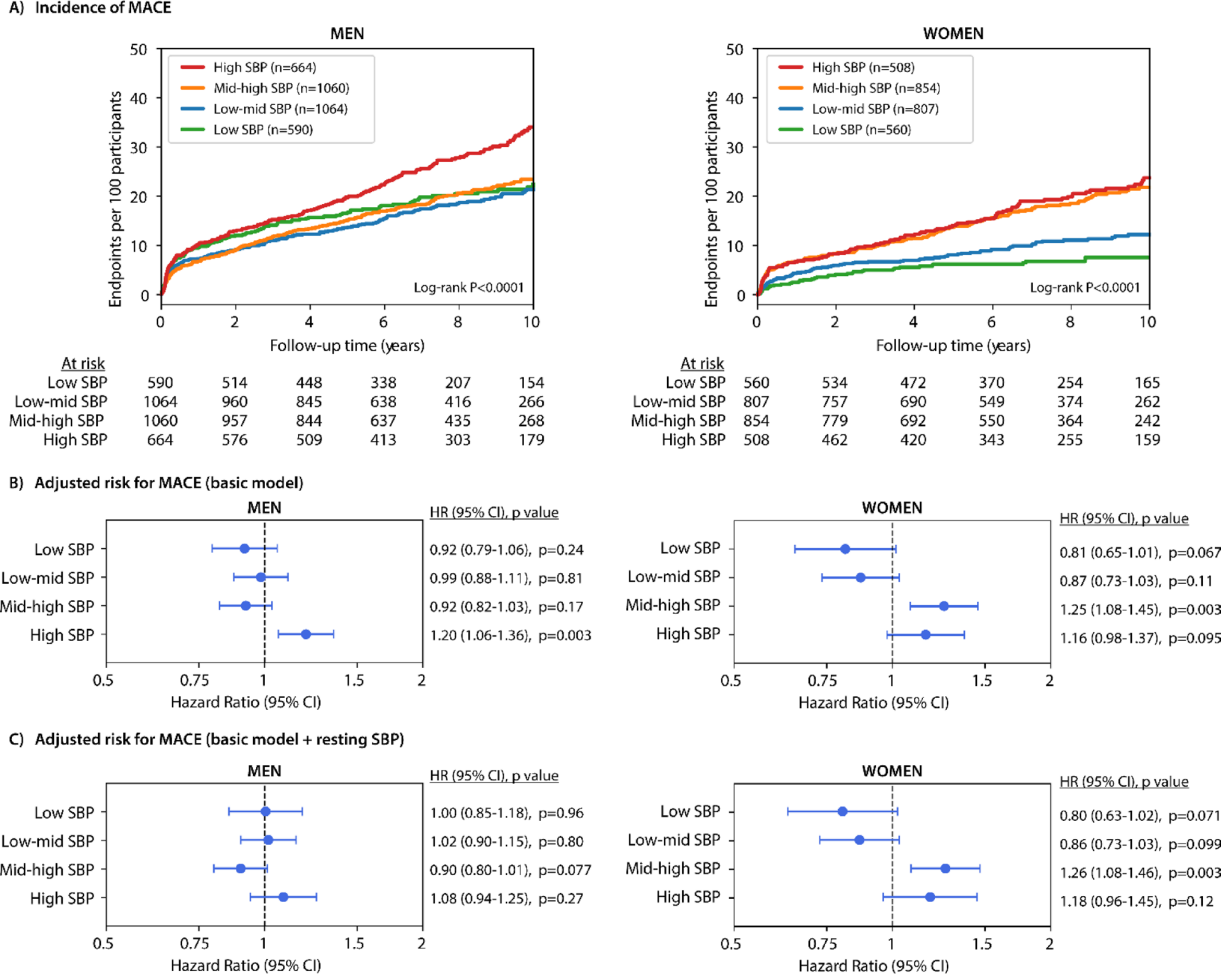
Risk of major adverse cardiovascular events (MACE) by SBP trajectories and sex. In **B** and **C**, hazard ratios (HR) are expressed relative to the cohort’s average risk. In** B** and** C**, HRs were adjusted for baseline age, cardiac disease, COPD, diabetes, antihypertensive medication, beta blocker use, lipid-lowering drugs, anticoagulants and ppWL and, in** C** only, for resting SBP.

In men, MACE incidence was highest in the high SBP group, both in unadjusted (*P*_LOG-RANK_ < 0.0001) and multivariable-adjusted analyses (Fig. 3A-B). However, this association was no longer significant after adjusting for resting SBP (Fig. 3C). In women, MACE incidence was higher in the mid-high and high SBP groups (*P*_Log-rank_ < 0.0001), but only the mid-high SBP group remained significant after adjustment (Fig. 3B-C).

Survival analyses on ∆SBP trajectories revealed a graded increase in MACE incidence from high to low ∆SBP rise (Supplemental Fig. 6A). A low ∆SBP rise was associated with increased MACE risk in men after basic adjustment (Supplemental Fig. 6B). However, this association lost significance when additionally adjusting for resting SBP (Supplemental Fig. 6C).

### Combinations of exercise SBP/∆SBP groups and MACE risk

Agreement between SBP and ∆SBP trajectory assignments was weak in both men (Cramer’s V = 0.065) and women (Cramer’s V = 0.056) (Supplemental Tables 6 and 7), suggesting the two metrics captured different, complementary components of the exercise SBP response.

**Fig. 4:**
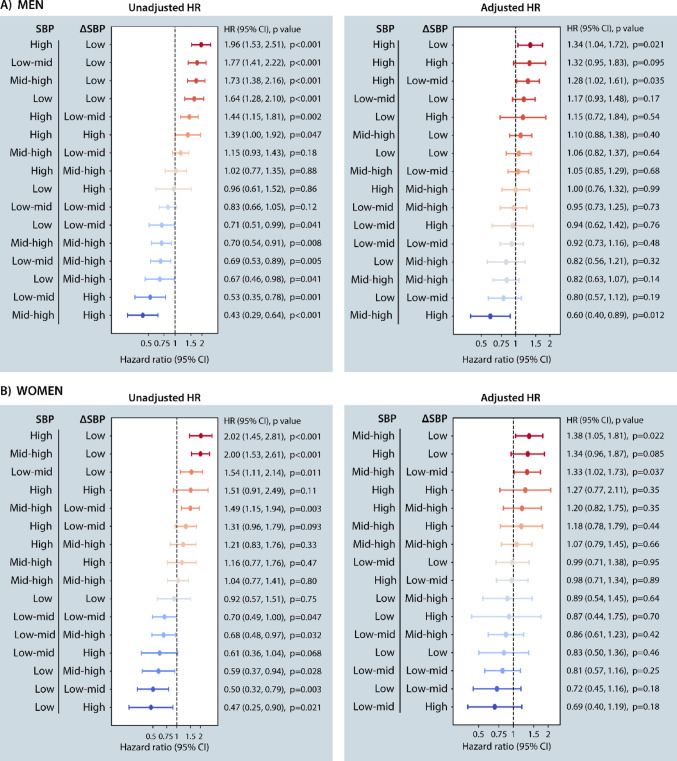
Risk of a major adverse cardiovascular event (MACE) by combinations of SBP/∆SBP trajectories in men (**A**) and women (**B**). Hazard ratios (HR) are expressed relative to the cohort’s average risk and were sorted accordingly. Adjusted HRs accounted for differences in significant predictors of MACE, i.e. baseline age, cardiac disease, COPD, diabetes, antihypertensive medication, beta blocker use, lipid-lowering drugs, anticoagulants and ppWL. Models with additional adjustment for resting SBP are presented in Supplemental Fig. 7.

In both unadjusted and adjusted analyses, men with high SBP/low ∆SBP consistently presented the highest risk of MACE (Fig. 4A). In women, those labelled as mid-high SBP/low(-mid) ∆SBP responder showed independently higher MACE risk (Fig. 4B, right panel). Additional adjustment for resting SBP attenuated the significance of these associations in men but not in women (Supplemental Fig. 7).

### Exercise (∆)SBP groups and MACE risk by hypertensive status

In women, normotension at rest was linked to the highest adjusted risk for MACE if presenting also a high SBP response (adjusted HR: 1.66 [1.06–2.61]), which exceeded even the risk associated with hypertension at rest (adjusted HRs between 0.94 and 1.30) (Supplemental Fig. 8). In men, a high SBP response was also associated with the highest adjusted risk for MACE, regardless if they were normotensive (1.23 [0.89, 1.70]) or hypertensive at rest (1.23 [1.05–1.43], Supplemental Fig. 8).

**Fig. 5:**
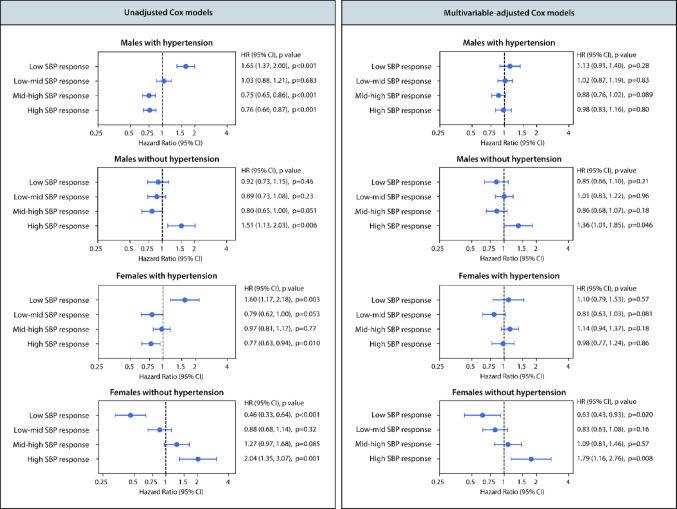
Risk of major adverse cardiovascular events (MACE) by SBP trajectories and sex, stratified by hypertensive status. Hazard ratios (HR) are expressed relative to the cohort’s average risk. Adjusted HRs accounted for differences in baseline age, cardiac disease, COPD, diabetes, antihypertensive medication (if hypertension at rest), beta blocker use, lipid-lowering drugs, anticoagulants, ppWL and resting SBP.

Within the group of men with normotension at rest, a high SBP response was independently associated with an increased risk of MACE (adjusted HR: 1.36 [1.01–1.85]; Fig. 5). Similarly, we observed an independent increase in MACE risk from low to high SBP responses in the group of normotensive women (adjusted HR for high SBP response: 1.79 [1.16–2.76]) (Fig. 5). In contrast, the SBP response groups did not predict MACE independently of clinical confounders within the groups of men and women with hypertension at rest (Fig. 5).

Regarding the ∆SBP responses, a low ∆SBP rise was independently associated with an increased MACE risk in male normotensive patients only (Supplemental Fig. 9).

## Discussion

We applied trajectory modelling to characterize sex-specific SBP responses during graded exercise testing (Graphical Abstract). The main findings were: (1) Four distinct trajectories emerged for each metric (SBP and ∆SBP), with weak concordance between them; (2) Both a high average SBP response (high SBP) and a blunted SBP rise (low ∆SBP) were linked to adverse cardiovascular risk profiles; (3) MACE incidence was highest in the high SBP and low ∆SBP groups, but these response categories did not independently predict MACE after adjusting for resting SBP; 4) In both sexes, the combination of a high average SBP response and a low SBP rise conferred the greatest MACE risk, though adjustment for resting SBP attenuated the significance of these associations; and 5) a high SBP response (along with a low ∆SBP in men) independently predicted future MACE among patients without hypertension at rest. Together, these findings underscore the potential of SBP trajectory analysis to refine cardiovascular risk stratification, particularly in individuals without preexisting hypertension.

### Analysing the full exercise SBP response for unbiased evaluation

Previous work on exercise SBP has mainly focused on discrete metrics such as peak SBP (Hedman et al. 2022; Assaf et al. 2021) and two-point SBP slopes (Hedman et al. 2019, 2022; Janssens et al. 2025) with demonstrated prognostic value. However, these simplified measures capture only part of the physiological response, potentially leading to oversimplification. The discrete metrics also rely on hard, arbitrary cutoffs for defining abnormality, which may introduce bias. In contrast, analysing the full SBP time-series through trajectory modelling enables an objective and comprehensive evaluation of the SBP dynamics during exercise. In the present study, individuals with similar peak values could be assigned to different trajectory groups, suggesting that trajectory-based classification captures heterogeneity in SBP dynamics that is not reflected by single-point measures alone.

With this regard, Nene et al. recently applied latent class analysis on 13,945 patients who underwent a treadmill testing with the Bruce protocol (Nene et al. 2025). From absolute SBP values measured at the three initial Bruce stages, three SBP trajectories were identified that reflect the mean SBP response (e.g. the high SBP responders were characterized by both high rest and high peak SBP) (Nene et al. 2025). Yet, these trajectories were not sex specific, resulting in male dominance in the higher SBP trajectories, and were limited to absolute exercise intensities within a workload range that was submaximal for many patients. Despite these limitations, their study is an important first step toward trajectory-driven evaluation of SBP dynamics during exercise.

In contrast to Nene et al (Nene et al. 2025), we identified SBP trajectories from a maximal, ramp-based exercise test, during which the gradual rise in workload ensures a smooth and gradual rise in SBP until maximal exertion is reached. This protocol yields physiologically richer SBP time-series, allowing relative comparison of SBP responses from rest to peak effort (instead of comparison between absolute exercise intensities at a set of fixed workload levels). Additionally, we eliminated potential sex bias by conducting the trajectory analyses for men and women separately. In both sexes, we identified response profiles that differed in the average SBP response (i.e. the SBP trajectories) and in SBP dynamics (i.e. the ∆SBP trajectories).

### Mean absolute versus relative SBP response: capturing distinct information

The two metrics thus seemed to capture distinct physiological information of the SBP response. The SBP trajectories predominantly grouped patients based on their mean exercise SBP, reflecting the average pressure load during exercise. This is similar to the SBP trajectories identified by Nene et al. (2025) The ∆SBP trajectories on the other hand grouped patients according to their relative rate of SBP increase (slope). Notably, the overlap between the SBP and ∆SBP classifications was low, observing a strong variety in ∆SBP responses within each SBP trajectory and vice versa. As such, a high average SBP response did not necessarily coincide with a steep (or low) SBP rise.

Both types of trajectory profiles were associated with baseline cardiovascular risk factors. In line with prior studies on peak SBP (Hedman et al. 2021, 2019; Nayor et al. 2023), a stronger average SBP response was associated with ageing, obesity, hypertension, and absence of beta blockers. Conversely, ageing and beta blockers were linked to a blunted rise in SBP (∆SBP), supporting prior findings on two-point SBP slopes (Hedman et al. 2019). Importantly, higher average SBP response and stronger SBP rise were both related to greater exercise capacity. This suggests that the absolute SBP values should be interpreted in the light of the relative SBP response and cardiopulmonary fitness, as fit individuals could be assigned to a high average SBP response because of their capability to achieve high workloads, requiring a large cardiac output and, subsequently, a high peak SBP (Nayor et al. 2023; Janssens et al. 2025). Together, these findings suggest that (∆)SBP trajectories reflect cardiovascular risk and (sub)clinical dysfunction but the extent to which they are mediated, confounded or moderated by the identified clinical factors warrants further investigation. Future studies should also explore how systemic regulatory mechanisms, such as vascular resistance, sympathetic activation and thermoregulation, could affect assignment to absolute and relative SBP trajectories (Rowell 1993).

### The predictive value of (∆)SBP response trajectories

Prior studies observed independent associations between cardiovascular outcomes and discrete exercise metrics such as peak SBP (Zafrir et al. 2022) and SBP slope (Hedman et al. 2019; Hedman et al. 2022). Nene et al. reported that trajectories characterized by elevated SBP during exercise carried higher MACE risk, although the survival analyses were unadjusted and may have been influenced by the male dominance in the high SBP trajectories (Nene et al. 2025). In our study, the exercise (∆)SBP responses were prognostically informative among patients who had no prior diagnosis of hypertension and were normotensive at rest, even after accounting for conventional risk factors including resting SBP. Our findings support the idea that, in normotensive individuals, abnormal exercise SBP patterns may unmask subclinical vascular stiffness, impaired vasodilatory reserve, or limited cardiac output augmentation that are not evident at rest but with adverse prognosis on the long run [Carlén et al. 2024, Tzemos et al. 2015]. Both excessive absolute pressure load during exercise and limited SBP responsiveness likely represent maladaptive hemodynamic phenotypes.

In contrast, the exercise SBP trajectories demonstrated no independent predictive value among patients with resting hypertension. Previously, Fagard et al. observed that, in a hypertensive cohort, exercise SBP did not significantly predict the incidence of MACE above resting SBP (Fagard et al. 1996). Adding to this observation, our findings suggest little to no incremental value for risk stratification by exercise hemodynamic responses beyond baseline BP burden in hypertensive cohorts. Future studies should deeper investigate why exercise SBP trajectories act as a risk discriminator mainly in normotensive individuals and how they may improve risk stratification in normotensive populations.

### Combining information from absolute and relative SBP response to enhance risk prediction

With the SBP trajectories reflecting the mean SBP response and the ∆SBP trajectories reflecting relative SBP responsiveness, they seem to capture different information from the hemodynamic response to exercise, with weak overlap between them. Combining this information may better characterize the SBP response and, as a result, refine risk prediction.

When combining trajectory assignments, the high SBP/low ∆SBP phenotype consistently conferred the highest MACE risk in both sexes. Although not independent from resting SBP, this combination likely represents the most maladaptive response, characterized by high pressure load throughout the test and limited capacity to further augment SBP with increasing workload. Integrating absolute and relative SBP trajectories may further improve risk stratification, particularly in normotensive cohorts, but due to extensive subgrouping such analyses require larger samples than the sample analysed here.

#### Clinical implications and perspectives

Our analytical approach enables qualitative classification of both the average and dynamic SBP response during graded exercise, providing a standardized and objective framework for interpretation. This approach minimizes reliance on arbitrary thresholds and may provide automated and reproducible characterization of SBP responses in clinical and research settings. Of note, the SBP trajectories may provide more granular phenotyping than the current binary classifications, as supported by the observation of graded differences in MACE risk across the SBP response categories, particularly among normotensive women. If validated prospectively, SBP trajectory profiling could be incorporated into exercise-testing software to automatically classify patients into distinct SBP response phenotypes, guiding cardiovascular risk stratification and clinical decision-making. Future studies should determine whether trajectory-based profiling improves disease prediction, diagnosis and prognosis beyond conventional risk factors and traditional peak SBP metrics and whether interventions targeting SBP dynamics can modify risk.

#### Strengths and limitations

We analysed a large, well-characterized cohort with systematic long-term follow-up and applied a robust framework for trajectory modelling. The algorithmic approach minimized subjectivity and enabled reproducible, sex-specific identification of SBP response patterns. However, several limitations must be acknowledged. First, as trajectories were derived from maximal clinical exercise tests using ramp protocols, the findings may not generalize to submaximal or stage-based protocols. Second, SBP measurements during exercise are prone to measurement error. Moreover, the variation in the timing and number of SBP measurements in the clinical exercise database may have influenced the precision of the trajectory modelling, despite applying thorough quality control, normalization and resampling procedures. Future work should assess whether standardized timing for SBP measurements improves reproducibility. Third, the requirement of at least four SBP measurements excludes tests with sparse SBP data, potentially omitting specific patient groups. Finally, the retrospective study design precludes causal inference and the clinical cohort may have underrepresented healthy individuals. Prospective validation in broader populations is therefore needed to confirm the generalizability of these findings.

#### Conclusions

We developed an algorithmic approach for objective assessment of absolute and relative SBP trajectories during maximal graded exercise. Distinct, sex-specific trajectories were identified, with both a high average SBP and a blunted SBP rise being associated with unfavourable cardiovascular risk profiles. In particular, a high SBP response (along with a low ∆SBP in men) independently predicted future MACE among patients who were normotensive at rest. These findings support the integration of SBP time-series analysis into exercise testing as a novel, physiologically grounded tool for refined cardiovascular risk stratification.

## Supplementary Information

Below is the link to the electronic supplementary material.

Supplementary Material 1

## Data Availability

The data underlying this article is available from the corresponding author upon reasonable request.

## Abbreviations

BIC: Bayesian Information Criterion
BMI: Body mass index
GBTM: Group-based trajectory modelling
MACE: Major adverse cardiovascular events
ppWL: Percent-predicted workload
SBP: Systolic blood pressure
∆SBP: Relative change in systolic blood pressure
