# Supplementary material for: Determinants and prognostic value of blood pressure trajectories during graded exercise
Source: Eur J Appl Physiol. 2026 May 8;126(8):4409–21. doi: 10.1007/s00421-026-06258-x (PMC13451356; doi:10.1007/s00421-026-06258-x)
Supplement: Supplementary file 1 [file 421_2026_6258_MOESM1_ESM.docx]

**Determinants and Prognostic Value of Blood Pressure Trajectories
During Graded Exercise**

Nicholas Cauwenberghs, Anna Carlén, Thomas Lindow, Viktor Elmberg, Lars Brudin,
Magnus Ekström, Kristofer Hedman

*Supplemental data*

**Supplemental Table 1. Clinical characteristics by sex.**

| **Variable** | **Women (n=2729)** | **Men (n=3378)** | ***P* value** |
| --- | --- | --- | --- |
| Age, yrs | 57.7±12.6 | 53.6±14.4 | <0.001 |
| Weight, kg | 72.3±12.5 | 87.0±13.2 | <0.001 |
| Length, cm | 165.2±5.9 | 179.2±6.5 | <0.001 |
| Body mass index, kg/m² | 26.5±4.3 | 27.1±3.7 | <0.001 |
| *Disease history and medication* |  |  |  |
| Hypertension, n (%) | 745 (27.3%) | 895 (26.5%) | 0.50 |
| Treated for hypertension, n (%) | 723 (26.5%) | 857 (25.4%) | 0.33 |
| Use of beta blockers, n (%) | 564 (20.7%) | 614 (18.2%) | 0.016 |
| Diabetes mellitus, n (%) | 117 (4.3%) | 220 (6.5%) | <0.001 |
| Treated for diabetes, n (%) | 101 (3.7%) | 200 (5.9%) | <0.001 |
| Lipid-lowering drugs, n (%) | 348 (12.8%) | 564 (16.7%) | <0.001 |
| Anticoagulants, n (%) | 377 (13.8%) | 589 (17.4%) | <0.001 |
| Renal failure, n (%) | 12 (0.4%) | 29 (0.9%) | 0.067 |
| COPD, n (%) | 24 (0.9%) | 18 (0.5%) | 0.14 |
| Heart failure, n (%) | 11 (0.4%) | 19 (0.6%) | 0.48 |
| Atrial fibrillation, n (%) | 57 (2.1%) | 110 (3.3%) | 0.007 |
| IHD before test, n (%) | 290 (10.6%) | 423 (12.5%) | 0.024 |
| Cardiac disease, n (%) | 342 (12.5%) | 524 (15.5%) | <0.001 |
| Cerebrovascular disease, n (%) | 20 (0.7%) | 43 (1.3%) | 0.051 |
| Cardiovascular disease, n (%) | 360 (13.2%) | 554 (16.4%) | <0.001 |
| *Exercise test data* |  |  |  |
| Resting SBP, mmHg | 132.7±21.2 | 130.8±18.9 | <0.001 |
| Resting heart rate, bpm | 81.2±13.7 | 79.9±14.3 | <0.001 |
| Peak SBP, mmHg | 191.0±24.4 | 202.5±24.4 | <0.001 |
| Peak heart rate, bpm | 154.7±19.7 | 159.5±21.3 | <0.001 |
| Exercise duration, min | 10.2±2.3 | 10.4±2.3 | <0.001 |
| Peak Borg, points | 17.4±0.8 | 17.6±0.9 | <0.001 |
| Peak workload, Watts | 134.5±26.6 | 214.4±46.2 | <0.001 |
| ppWL, % | 97.4±14.7 | 93.5±14.6 | <0.001 |
| Values are mean±SD or count (frequency).bpm, beats per minute; COPD, chronic obstructive pulmonary disease; IHD, ischemic heart disease; ppWL, percentage predicted workload; SBP, systolic blood pressure. | | | |

**Supplemental Table 2. Concordance between exaggerated SBP response and the (∆)SBP trajectory assignments by sex.** Exaggerated SBP response was defined as peak SBP >210 mmHg in men and >190 mmHg in women.

| **(∆)SBP trajectory by sex** | **Exaggerated SBP response** | |
| --- | --- | --- |
|  | No | Yes |
| *SBP trajectories in men (Cramer’s V=0.73)* |  |  |
| Low SBP response | 589 (17.4%) | 1 (0.0%) |
| Low-mid SBP response | 1027 (30.4%) | 37 (1.1%) |
| Mid-high SBP response | 626 (18.5%) | 434 (12.8%) |
| High SBP response | 52 (1.5%) | 612 (18.1%) |
| *SBP trajectories in women (Cramer’s V=0.76)* |  |  |
| Low SBP response | 558 (20.4%) | 2 (0.1%) |
| Low-mid SBP response | 668 (24.5%) | 139 (5.1%) |
| Mid-high SBP response | 217 (8.0%) | 637 (23.3%) |
| High SBP response | 4 (0.1%) | 504 (18.5%) |
| *∆SBP trajectories in men (Cramer’s V=0.19)* |  |  |
| Low SBP risers | 606 (17.9%) | 140 (4.1%) |
| Low-mid SBP risers | 780 (23.1%) | 330 (9.8%) |
| Mid-high SBP risers | 620 (18.4%) | 375 (11.1%) |
| High SBP risers | 288 (8.5%) | 239 (7.1%) |
| *∆SBP trajectories in women (Cramer’s V=0.13)* |  |  |
| Low SBP risers | 372 (13.6%) | 212 (7.8%) |
| Low-mid SBP risers | 495 (18.1%) | 426 (15.6%) |
| Mid-high SBP risers | 398 (14.6%) | 427 (15.6%) |
| High SBP risers | 182 (6.7%) | 217 (8.0%) |

**Supplemental Table 3 . Clinical characteristics by SBP trajectories and sex.**

| **Variable** | **Men** | | | | **Women** | | | |
| --- | --- | --- | --- | --- | --- | --- | --- | --- |
|  | Low SBP (n=590) | Low-mid SBP (n=1064) | Mid-high SBP (n=1060) | High SBP (n=664) | Low SBP (n=560) | Low-mid SBP (n=807) | Mid-high SBP (n=854) | High SBP (n=508) |
| Age, yrs | 50.0±15.5 | 50.3±15.1 | 55.4±13.2*† | 58.9±11.4*†‡ | 48.3±13.9 | 56.7±12.2* | 61.2±10.1*† | 64.0±8.7*†‡ |
| Weight, kg | 84.2±13.5 | 86.7±13.1* | 87.5±13.1* | 89.0±12.9*†‡ | 69.3±12.6 | 71.1±12.1* | 73.9±12.1*† | 74.9±12.9*† |
| Length, cm | 179.0±6.7 | 179.8±6.6* | 179.3±6.5 | 178.3±6.3†‡ | 166.4±6.1 | 165.0±5.9* | 164.9±5.9* | 164.7±5.5* |
| Body mass index, kg/m² | 26.3±3.8 | 26.8±3.7* | 27.2±3.6*† | 28.0±3.6*†‡ | 25.0±4.3 | 26.1±4.1* | 27.1±4.1*† | 27.6±4.5*†‡ |
| *Diseases and medication* |  |  |  |  |  |  |  |  |
| Hypertension, n (%) | 120 (20.3%) | 210 (19.7%) | 301 (28.4%)*† | 264 (39.8%)*†‡ | 54 (9.6%) | 176 (21.8%)* | 294 (34.4%)*† | 221 (43.5%)*†‡ |
| Treated for hypertension, n (%) | 113 (19.2%) | 201 (18.9%) | 289 (27.3%)*† | 254 (38.3%)*†‡ | 51 (9.1%) | 171 (21.2%)* | 284 (33.3%)*† | 217 (42.7%)*†‡ |
| Use of beta blockers, n (%) | 136 (23.1%) | 185 (17.4%)* | 183 (17.3%)* | 110 (16.6%)* | 88 (15.7%) | 147 (18.2%) | 207 (24.2%)*† | 122 (24.0%)*† |
| Diabetes mellitus, n (%) | 35 (5.9%) | 58 (5.5%) | 70 (6.6%) | 57 (8.6%)† | 9 (1.6%) | 36 (4.5%)* | 37 (4.3%)* | 35 (6.9%)* |
| Treated for diabetes, n (%) | 31 (5.3%) | 52 (4.9%) | 67 (6.3%) | 50 (7.5%)† | 8 (1.4%) | 30 (3.7%)* | 32 (3.7%)* | 31 (6.1%)* |
| Lipid-lowering drugs, n (%) | 103 (17.5%) | 164 (15.4%) | 166 (15.7%) | 131 (19.7%)†‡ | 38 (6.8%) | 92 (11.4%)* | 136 (15.9%)*† | 82 (16.1%)*† |
| Anticoagulants, n (%) | 134 (22.7%) | 181 (17.0%)* | 162 (15.3%)* | 112 (16.9%)* | 56 (10.0%) | 109 (13.5%) | 139 (16.3%)* | 73 (14.4%)* |
| Renal failure, n (%) | 7 (1.2%) | 6 (0.6%) | 10 (0.9%) | 6 (0.9%) | 3 (0.5%) | 6 (0.7%) | 2 (0.2%) | 1 (0.2%) |
| COPD, n (%) | 4 (0.7%) | 1 (0.1%) | 4 (0.4%) | 9 (1.4%)†‡ | 3 (0.5%) | 6 (0.7%) | 10 (1.2%) | 5 (1.0%) |
| Heart failure, n (%) | 4 (0.7%) | 4 (0.4%) | 8 (0.8%) | 3 (0.5%) | 4 (0.7%) | 4 (0.5%) | 1 (0.1%) | 2 (0.4%) |
| Atrial fibrillation, n (%) | 27.0 (4.6%) | 37.0 (3.5%) | 36.0 (3.4%) | 10.0 (1.5%)*†‡ | 15.0 (2.7%) | 19.0 (2.4%) | 17.0 (2.0%) | 6.0 (1.2%) |
| IHD before test, n (%) | 92 (15.6%) | 127 (11.9%)* | 120 (11.3%)* | 84 (12.7%) | 33 (5.9%) | 91 (11.3%)* | 114 (13.3%)* | 52 (10.2%)* |
| Cardiac disease, n (%) | 117 (19.8%) | 154 (14.5%)* | 158 (14.9%)* | 95 (14.3%)* | 47 (8.4%) | 107 (13.3%)* | 129 (15.1%)* | 59 (11.6%) |
| Cerebrovascular disease, n (%) | 6 (1.0%) | 13 (1.2%) | 12 (1.1%) | 12 (1.8%) | 2 (0.4%) | 8 (1.0%) | 4 (0.5%) | 6 (1.2%) |
| Cardiovascular disease, n (%) | 119 (20.2%) | 163 (15.3%)* | 167 (15.8%)* | 105 (15.8%) | 49 (8.8%) | 114 (14.1%)* | 132 (15.5%)* | 65 (12.8%)* |
| *Exercise test data* |  |  |  |  |  |  |  |  |
| Resting SBP, mmHg | 111.2±12.1 | 123.12±11.6* | 135.69±13.3*† | 152.7±15.0*†‡ | 108.6±11.3 | 124.4±11.6* | 140.7±13.4*† | 159.2±13.8*†‡ |
| Resting heart rate, bpm | 79.3±14.2 | 80.15±13.7 | 79.94±14.8 | 80.2±14.3 | 81.6±14.0 | 81.1±14.0 | 81.0±13.8 | 81.5±12.7 |
| Peak SBP, mmHg | 170.1±14.4 | 192.53±11.2* | 211.05±11.8*† | 233.5±15.9*†‡ | 160.5±13.0 | 181.7±10.8* | 200.4±11.7*† | 223.5±15.1*†‡ |
| Peak heart rate, bpm | 156.4±24.0 | 162.06±21.4* | 159.39±21.0*† | 158.1±18.5† | 159.4±21.0 | 155.0±20.3* | 153.1±18.9* | 151.75±17.9*† |
| Exercise duration, min | 9.9±2.3 | 10.70±2.3* | 10.51±2.3* | 10.2±2.2*†‡ | 10.8±2.3 | 10.4±2.3* | 10.0±2.3*† | 9.5±2.1*†‡ |
| Peak Borg, points | 17.6±0.9 | 17.65±0.9 | 17.64±0.9 | 17.5±0.8†‡ | 17.6±0.9 | 17.4±0.8* | 17.4± 0.7* | 17.3±0.7*† |
| Peak workload, watts | 203.7±42.1 | 220.36±45.3* | 218.13±48.4* | 208.2±45.1†‡ | 142.7±27.0 | 137.5±27.9* | 131.4±25.7*† | 125.9±21.9*†‡ |
| ppWL, % | 86.8±14.2 | 92.92±14.0* | 96.13±14.3*† | 96.2±14.4*† | 93.2±14.4 | 98.5±15.2* | 98.7± 14.6* | 98.0±13.6* |
| Values are mean±SD or count (frequency). SBP, systolic blood pressure; COPD, chronic obstructive pulmonary disease; IHD, ischemic heart disease; ppWL, percentage predicted workload.  Statistical comparisons: *p<0.05 versus low SBP, †p<0.05 versus low-mid, ‡p<0.05 versus mid-high SBP. | | | | | | | | |

**Supplemental Table 4. Clinical characteristics by ∆SBP trajectories and sex.**

| **Variable** | **Men** | | | | **Women** | | | |
| --- | --- | --- | --- | --- | --- | --- | --- | --- |
|  | Low risers (n=746) | Low-mid risers (n=1110) | Mid-high risers (n=995) | High risers (n=527) | Low risers (n=584) | Low-mid risers (n=921) | Mid-high risers (n=825) | High risers (n=399) |
| Age, yrs | 59.2±13.3 | 53.7±14.4* | 51.2±14.0*† | 49.7±13.9*†‡ | 61.9±13.1 | 57.4±12.9* | 56.2±11.5*† | 55.6±12.4*† |
| Weight, kg | 87.6±13.8 | 86.9±13.4 | 87.2±12.9 | 85.8±12.6*‡ | 72.5±12.6 | 72.8±12.8 | 72.1±12.4 | 71.4±12.1 |
| Length, cm | 178.3±6.5 | 179.0±6.5* | 179.6±6.4*† | 180.0±6.8*† | 164.5±5.8 | 165.3±5.8* | 165.4±6.1* | 165.8±5.9* |
| Body mass index, kg/m² | 27.5±3.9 | 27.1±3.8* | 27.0±3.6* | 26.5±3.5*†‡ | 26.8±4.4 | 26.7±4.5 | 26.3±4.2 | 26.0±4.1*† |
| *Diseases and medication* |  |  |  |  |  |  |  |  |
| Hypertension, n (%) | 278 (37.3%) | 304 (27.4%)* | 218 (21.9%)*† | 95 (18.0%)*† | 247 (42.3%) | 257 (27.9%)* | 167 (20.2%)*† | 74 (18.5%)*† |
| Treated for hypertension, n (%) | 261 (35.0%) | 298 (26.8%)* | 210 (21.1%)*† | 88 (16.7%)*†‡ | 237 (40.6%) | 251 (27.3%)* | 164 (19.9%)*† | 71 (17.8%)*† |
| Use of beta blockers, n (%) | 242 (32.4%) | 199 (17.9%)* | 120 (12.1%)*† | 53 (10.1%)*† | 223 (38.2%) | 192 (20.8%)* | 106 (12.8%)*† | 43 (10.8%)*† |
| Diabetes mellitus, n (%) | 71 (9.5%) | 81 (7.3%) | 43 (4.3%)*† | 25 (4.7%)* | 21 (3.6%) | 41 (4.5%) | 26 (3.2%) | 29 (7.3%)*†‡ |
| Treated for diabetes, n (%) | 66 (8.8%) | 71 (6.4%) | 40 (4.0%)*† | 23 (4.4%)* | 18 (3.1%) | 33 (3.6%) | 23 (2.8%) | 27 (6.8%)*†‡ |
| Lipid-lowering drugs, n (%) | 167 (22.4%) | 192 (17.3%)* | 137 (13.8%)*† | 68 (12.9%)*† | 105 (18.0%) | 107 (11.6%)* | 84 (10.2%)* | 52 (13.0%)* |
| Anticoagulants, n (%) | 213 (28.6%) | 179 (16.1%)* | 135 (13.6%)* | 62 (11.8%)*† | 126 (21.6%) | 121 (13.1%)* | 82 (9.9%)*† | 48 (12.0%)* |
| Renal failure, n (%) | 11 (1.5%) | 10 (0.9%) | 5 (0.5%) | 3 (0.6%) | 8 (1.4%) | 1 (0.1%)* | 2 (0.2%)* | 1 (0.3%) |
| COPD, n (%) | 4 (0.5%) | 8 (0.7%) | 1 (0.1%) | 5 (0.9%)‡ | 9 (1.5%) | 6 (0.7%) | 6 (0.7%) | 3 (0.8%) |
| Heart failure, n (%) | 10 (1.3%) | 4 (0.4%)* | 3 (0.3%)* | 2 (0.4%) | 6 (1.0%) | 4 (0.4%) | 1 (0.1%)* | 0 (0.0%) |
| Atrial fibrillation, n (%) | 40.0 (5.4%) | 34.0 (3.1%)* | 24.0 (2.4%)* | 12.0 (2.3%)* | 26.0 (4.5%) | 18.0 (2.0%)* | 6.0 (0.7%)*† | 7.0 (1.8%)* |
| IHD before test, n (%) | 156 (20.9%) | 132 (11.9%)* | 96 (9.6%)* | 39 (7.4%)*† | 83 (14.2%) | 94 (10.2%)* | 75 (9.1%)* | 38 (9.5%)* |
| Cardiac disease, n (%) | 187 (25.1%) | 165 (14.9%)* | 121 (12.2%)* | 51 (9.7%)*† | 110 (18.8%) | 110 (11.9%)* | 79 (9.6%)* | 43 (10.8%)* |
| Cerebrovascular disease, n (%) | 13 (1.7%) | 12 (1.1%) | 10 (1.0%) | 8 (1.5%) | 3 (0.5%) | 6 (0.7%) | 7 (0.8%) | 4 (1.0%) |
| Cardiovascular disease, n (%) | 193 (25.9%) | 174 (15.7%)* | 129 (13.0%)* | 58 (11.0%)*† | 112 (19.2%) | 116 (12.6%)* | 86 (10.4%)* | 46 (11.5%)* |
| *Exercise test data* |  |  |  |  |  |  |  |  |
| Resting SBP, mmHg | 145.4±18.4 | 134.4±16.0* | 124.7±14.6*† | 114.1±13.7*†‡ | 149.5±18.8 | 136.9±18.7* | 125.6±16.5*† | 113.1±15.6*†‡ |
| Resting heart rate, bpm | 79.3±16.3 | 80.6±14.0 | 80.6±13.8 | 78.8±12.6†‡ | 79.1±14.9 | 81.3±13.2* | 82.7±13.4* | 81.6±13.2* |
| Peak SBP, mmHg | 191.6±24.6 | 201.7±23.1* | 206.6±23.2*† | 211.8±23.1*†‡ | 184.3±22.2 | 190.3±24.7* | 193.5±24.2*† | 197.0±24.9*†‡ |
| Peak heart rate, bpm | 149.0±23.0 | 159.3±20.8* | 163.8±19.3*† | 166.6±17.5*†‡ | 145.3±22.4 | 154.6±19.3* | 158.8±16.76*† | 160.6±17.0*† |
| Exercise duration, min | 9.4±2.2 | 10.3±2.2* | 10.8±2.3*† | 11.2±2.2*†‡ | 9.3±2.1 | 10.2±2.3* | 10.5±2.3*† | 10.8±2.3*†‡ |
| Peak Borg, points | 17.5±0.8 | 17.6±0.9* | 17.7±0.9* | 17.7±0.9*† | 17.4±0.7 | 17.4±0.8 | 17.4±0.8 | 17.5±0.8*† |
| Peak workload, watts | 192.0±39.1 | 211.2±42.9* | 223.8±45.7*† | 235.0±48.8*†‡ | 122.8±21.7 | 134.4±25.8* | 138.3±26.4*† | 144.2±29.2*†‡ |
| ppWL, % | 90.1±14.4 | 92.7±14.3* | 94.9±14.4*† | 97.6±14.7*†‡ | 94.3±13.6 | 97.1±14.9* | 98.0±14.7* | 101.2±15.0*†‡ |
| Values are mean±SD or count (frequency). SBP, systolic blood pressure; COPD, chronic obstructive pulmonary disease; IHD, ischemic heart disease; ppWL, percentage predicted workload.  Statistical comparisons: *p<0.05 versus low SBP, †p<0.05 versus low-mid, ‡p<0.05 versus mid-high SBP. | | | | | | | | |

**Supplemental Table 5. Event rates for major adverse cardiovascular events by SBP and ∆SBP trajectories and sex.**

|  | Men | | | Women | | |
| --- | --- | --- | --- | --- | --- | --- |
| Group | MACE | | MACE per 1000 person-years | MACE | | MACE per 1000 person-years |
| Whole sample | 755 | | 31.1 |  | |  |
| By SBP trajectories | |  | | |  | |
| Low SBP | 122 | | 29.8 | 42 | | 9.6 |
| Low-mid SBP | 202 | | 26.4 | 93 | | 14.5 |
| Mid-high SBP | 222 | | 28.9 | 169 | | 26.4 |
| High SBP | 209 | | 42.9 | 108 | | 27.0 |
| By ∆SBP trajectories |  | |  |  | |  |
| Low SBP risers | 267 | | 55.2 | 136 | | 31.8 |
| Low -mid SBP risers | 248 | | 30.6 | 130 | | 18.0 |
| Mid-high SBP risers | 161 | | 21.7 | 100 | | 15.0 |
| High SBP risers | 79 | | 19.9 | 46 | | 15.3 |
| Incidence rates were calculated as number of events per 1000 subject-years. | | | | | | |

**Supplemental Table 6. Concordance between SBP and ∆SBP trajectories in women.** Cramer’s V tests indicated a very weak association between the SBP and ∆SBP trajectory assignments (Cramer’s V= 0.056).

|  |  | | **∆SBP trajectory** | | | |  |
| --- | --- | --- | --- | --- | --- | --- | --- |
|  |  | Low risers | | Low-mid risers | Mid-high risers | High risers | Total |
| **SBP trajectory** | Low SBP | 104 (3.8%) | | 196 (7.2%) | 162 (5.9%) | 98 (3.6%) | 560 (20.5%) |
|  | Low-mid SBP | 153 (5.6%) | | 257 (9.4%) | 275 (10.1%) | 122 (4.5%) | 807 (29.6%) |
|  | Mid-high SBP | 207 (7.6%) | | 276 (10.1%) | 252 (9.2%) | 119 (4.4%) | 854 (31.3%) |
|  | High SBP | 120 (4.4%) | | 192 (7.0%) | 136 (5.0%) | 60 (2.2%) | 508 (18.6%) |
|  | Total | 584 (21.4%) | | 921 (33.7%) | 825 (30.2%) | 399 (14.6%) | 2729 (100.0%) |

**Supplemental Table 7. Concordance between SBP and ∆SBP trajectories in men.** Cramer’s V tests indicated a very weak association between the SBP and ∆SBP trajectory assignments (Cramer’s V=0.065).

|  |  | | **∆SBP trajectory** | | | |  |
| --- | --- | --- | --- | --- | --- | --- | --- |
|  |  | Low risers | | Low-mid risers | Mid-high risers | High risers | Total |
| **SBP trajectory** | Low SBP | 185 (5.5%) | | 182 (5.4%) | 149 (4.4%) | 74 (2.2%) | 590 (17.5%) |
|  | Low-mid SBP | 205 (6.1%) | | 371 (11.0%) | 328 (9.7%) | 160 (4.7%) | 1064 (31.5%) |
|  | Mid-high SBP | 211 (6.2%) | | 336 (9.9%) | 324 (9.6%) | 189 (5.6%) | 1060 (31.4%) |
|  | High SBP | 145 (4.3%) | | 221 (6.5%) | 194 (5.7%) | 104 (3.1%) | 664 (19.7%) |
|  | Total | 746 (22.1%) | | 1110 (32.9%) | 995 (29.5%) | 527 (15.6%) | 3378 (100.0%) |

**Supplemental Figure 1. Study flow chart.**

**Supplemental Figure 2. BIC plots to determine the optimal number of SBP and ∆SBP trajectories by sex.** In both men and women, the preferred number of trajectories for both SBP and ∆SBP was four or five, as indicated by the elbow-shaped hinge point on the curves. For consistency and to balance interpretability and granularity, we derived four trajectories for SBP and ∆SBP in both sexes.

**Supplemental Figure 3. Exercise SBP trajectories derived by group-based trajectory modelling (GBTM).** GBTM identified four distinct response profiles from the SBP time-series in men and women. Each panel shows the individual SBP time-series of the patients assigned to the trajectory (in grey) as well as the trajectory regression line (bold line).

**Supplemental Figure 4. Exercise ∆SBP trajectories derived by group-based trajectory modelling (GBTM).** GBTM identified four distinct response profiles from the ∆SBP time-series in men and women. Each panel shows the individual ∆SBP time-series of the patients assigned to the trajectory (in grey) as well as the trajectory regression line (bold line).

**Supplemental Figure 5. Peak SBP metrics by SBP (A) and ∆SBP (B) trajectories and sex.** Values on the plot represent the percentage overlap between adjacent distributions, indicating substantial overlap in peak SBP and peak ΔSBP distributions across the SBP and peak ΔSBP response groups, respectively.

**Supplemental Figure 6. Risk of major adverse cardiovascular events (MACE) by ∆SBP trajectories and sex.** In B and C, hazard ratios (HR) are expressed relative to the cohort’s average risk. In B and C, HRs were adjusted for baseline age, cardiac disease, COPD, diabetes, antihypertensive medication, beta blocker use, lipid-lowering drugs, anticoagulants and ppWL and, for C only, additionally for resting SBP.

**Supplemental Figure 7. Risk for a major adverse cardiovascular event (MACE) by combinations of SBP/∆SBP trajectories in men (left) and women (right) accounting for resting SBP.** Hazard ratios (HR) are expressed relative to the cohort’s average risk. Adjusted HRs accounted for differences in baseline age, cardiac disease, COPD, diabetes, antihypertensive medication, beta blocker use, lipid-lowering drugs, anticoagulants, ppWL and resting SBP.

**Supplemental Figure 8. Risk of major adverse cardiovascular events (MACE) by SBP trajectories, sex and hypertensive status.** Hazard ratios (HR) are expressed relative to the cohort’s average risk. Adjusted HRs accounted for differences in baseline age, cardiac disease, COPD, diabetes, beta blocker use, lipid-lowering drugs, anticoagulants, ppWL and resting SBP.

**Supplemental Figure 9. Risk of major adverse cardiovascular events (MACE) by ∆SBP trajectories and sex, stratified by hypertensive status.** Hazard ratios (HR) are expressed relative to the cohort’s average risk. Adjusted HRs accounted for differences in baseline age, cardiac disease, COPD, diabetes, antihypertensive medication (if hypertension at rest), beta blocker use, lipid-lowering drugs, anticoagulants, ppWL and resting SBP.
